# The Complex Proteolipidic Behavior of the SARS-CoV-2 Envelope Protein Channel: Weak Selectivity and Heterogeneous Oligomerization

**DOI:** 10.3390/ijms241512454

**Published:** 2023-08-05

**Authors:** Wahyu Surya, Ernesto Tavares-Neto, Andrea Sanchis, María Queralt-Martín, Antonio Alcaraz, Jaume Torres, Vicente M. Aguilella

**Affiliations:** 1 School of Biological Sciences, Nanyang Technological University, 60 Nanyang Drive, Singapore 637551, Singapore; wsurya@ntu.edu.sg; 2 Laboratory of Molecular Biophysics, Department of Physics, Universitat Jaume I, 12080 Castellon, Spain; tavaresn@uji.es (E.T.-N.); mqueralt@uji.es (M.Q.-M.); alcaraza@uji.es (A.A.)

**Keywords:** analytical ultracentrifugation, envelope protein, COVID-19, SARS-CoV, oligomerization, ion channel

## Abstract

The envelope (E) protein is a small polypeptide that can form ion channels in coronaviruses. In SARS coronavirus 2 (SARS-CoV-2), the agent that caused the recent COVID-19 pandemic, and its predecessor SARS-CoV-1, E protein is found in the endoplasmic reticulum–Golgi intermediate compartment (ERGIC), where virion budding takes place. Several reports claim that E protein promotes the formation of “cation-selective channels”. However, whether this term represents specificity to certain ions (e.g., potassium or calcium) or the partial or total exclusion of anions is debatable. Herein, we discuss this claim based on the available data for SARS-CoV-1 and -2 E and on new experiments performed using the untagged full-length E protein from SARS-CoV-2 in planar lipid membranes of different types, including those that closely mimic the ERGIC membrane composition. We provide evidence that the selectivity of the E-induced channels is very mild and depends strongly on lipid environment. Thus, despite past and recent claims, we found no indication that the E protein forms cation-selective channels that prevent anion transport, and even less that E protein forms *bona fide* specific calcium channels. In fact, the E channel maintains its multi-ionic non-specific neutral character even in concentrated solutions of Ca^2+^ ions. Also, in contrast to previous studies, we found no evidence that SARS-CoV-2 E channel activation requires a particular voltage, high calcium concentrations or low pH, in agreement with available data from SARS-CoV-1 E. In addition, sedimentation velocity experiments suggest that the E channel population is mostly pentameric, but very dynamic and probably heterogeneous, consistent with the broad distribution of conductance values typically found in electrophysiological experiments. The latter has been explained by the presence of proteolipidic channel structures.

## 1. Introduction

The SARS coronavirus 2 (SARS-CoV-2, or SARS-2 hereafter) is the causative agent of the COVID-19 pandemic, which so far has claimed almost seven million lives worldwide. Although vaccines against this viral infection are now widely available, therapeutic treatments for infected individuals are only beginning to emerge. One potential antiviral drug target is the channel formed by the envelope (E) protein [1]. E protein localizes to the endoplasmic reticulum–Golgi intermediate compartment (ERGIC) of infected cells [2]. In SARS-2, the E protein is 75 residues long (76 in SARS-1) and has a single transmembrane (TM) domain, with N-terminal and a C-terminal tails exposed lumenally and cytoplasmically, respectively. The N-terminal tail is approximately 10 residues long, whereas the C-terminal tail spans residues 39–75. The helical core of the TM domain (E-TM) encompasses residues 14–34, with residues 8–13 being more dynamic and exposed to water [3]. The E-TM domain forms oligomers with ion channel activity [4,5,6,7,8,9,10,11,12]. In the more studied SARS-CoV-1 E protein (SARS-1 E hereafter), this channel activity is a virulence factor, as shown by the effect of channel-inactivating mutations [4] on a mouse-adapted SARS-1 virus. The effect of these mutations was to reduce lung edema and proinflammatory cytokine levels [6].

**Figure 1:**
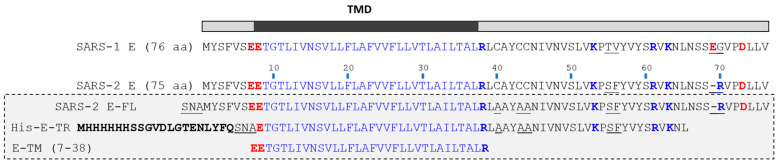
**Comparison of SARS E sequences.** Sequence comparison between SARS-1 E (accession number P59637) and SARS-2 E (accession number QHZ00401). Transmembrane domain (TMD) is shown in blue. SARS-2 E only differs from SARS-1 E at three mutations and one deletion (underlined in SARS-1 E sequence). The samples used in the present paper (gray rectangle) correspond to SARS-2: full length (E-FL), truncated with a His-tag (His-E-TR) or E-TM. Both E-FL and E-TR contain additional three N-terminal residues (SNA) arising from the N-terminal fusion tag that was cleaved in E-FL, and native Cys residues were mutated to Ala (underlined). For reference, charged residues are indicated as blue (positive) or red (negative) in bold.

The biology, and especially the channel properties, of E protein in SARS-1 and SARS-2 are likely to be very similar since they share an identical TM domain and they only differ in three rather conservative mutations and one deletion, all localized in the C-terminal domain (Figure 1). The channel-forming domain (E-TM) forms pentameric oligomers in perfluorooctanoic acid (PFO) gels [8] and ERGIC-like lipid membranes, as shown using ^19^F spin diffusion solid-state NMR [13]. Full-length (FL) SARS-1 protein (E-FL) was also pentameric in PFO gels and in C14 betaine detergent micelles tested using equilibrium sedimentation [7,9], and SARS-2 E-FL sedimentation behavior was consistent with pentamers in sedimentation velocity experiments [14]. More recently, the mass difference between lipidic nanodiscs reconstituted with SARS-2 E-FL protein and empty ones was consistent with an E protein pentameric oligomer [15].

E-TM is α-helical in both detergent and lipids [3,16,17]. A high-resolution model of the E-TM channel was determined recently in ERGIC-like membranes using solid-state NMR spectroscopy [3]. The channel lumen was dehydrated and described as being in a closed conformation, with a radius of 2 Å in the narrowest section, consistent with a low conducting channel. A pore-facing asparagine (Asn15) was in agreement with previous structural models [16,17] and with the electrophysiological findings that mutation N15A completely abolishes channel activity [4,11]. Studies using ^13^C and ^19^F solid-state NMR spectroscopy reported that low pH and high calcium concentration, supposed to mimic the ERGIC and lysosomal environment experienced by the E protein in the cell, produced a more open conformation of the channel [18], with conformational changes in TM N- and C-terminal residues.

The C-terminal domain is α-helical in detergent [9,19,20] but it forms β-structure in presence of lipids [9,14,21] possibly triggered by oligomerization, and a triple-stranded β-sheet that oligomerizes into a barrel-like structure outside the membrane has been described [21]. These interlocking β-strands may add stability to the pentameric form, compared to the pentamers formed by E-TM alone.

However, the fact that the channel structure reported [3] was rigid and closed, with no significant lipid involvement, requiring the presence of either calcium or low pH to produce a more open conformation [18], does not appear to be supported by functional evidence. First, the vast majority of electrophysiological experiments reported with the SARS-1 E channel did not involve acidification or require calcium to produce fully functional and open channels [4,5,8,10,11,12,16]. In fact, both low pH and addition of calcium over salts of monovalent cations significantly *reduced* the channel conductance [22]. Second, in experiments where E channel activity was directly correlated with virus pathogenesis in mice [6], weakly selective pores modulated by membrane lipid charge were reported for both SARS-1 E-FL and E-TM [12,23]. This strongly suggests a key role of lipids in E channel conformation.

More recent experiments with SARS-2 E protein support that this channel can transport different types of ions (Na^+^, K^+^, Ca^2+^, Cl^−^) with only mild selectivity. For example, mild ion selectivity in salts of monovalent cations were reported when SARS-2 E protein was expressed in intracellular organelles and the plasma membrane of mammalian cells and *Xenopus* oocytes [24]. Breitinger et al. also showed activity of SARS-2 E expressed in cells, reporting currents at different pHs and concluding that E was likely not a proton-gated channel [25]. In stark contrast, other recent papers [26] claim that SARS-2 E-FL forms pH-sensitive ideal cation-selective channels. However, based on the information provided, some of these results have difficult interpretation. For example, conductance is 50% higher in NaCl than in KCl. More problematic is that the reversal potential (RP) measured for 50/500 mM KCl (activity ratio ~8.3) exceeds the K^+^ Nernst potential. This impossible “beyond ideal” cation selectivity contrasts with the small RP reported by these authors for CaCl_2_ and MgCl_2_. The latter is consistent with the RP of a neutral channel with a weakly selective pore. Indeed, these values are very close to their respective diffusion potentials, V_diff_, caused by the different diffusion coefficients of cations and anions [27]. In another work (still a preprint at the time of submission of the present manuscript) [28], measurements with a His-tagged SARS-2 E channel in CaCl_2_ solutions are used to claim that the channel is activated by calcium and voltage. However, the stated conductance of ~12 pS in 510/210 mM CaCl_2_ is surprisingly low whereas the RP is about −19 mV, which again exceeds the Ca^2+^ Nernst potential. In addition, Poggio et al. also reported conductance values that are nearly one-third of the ones measured under similar conditions in SARS-1 E-FL [15], although in this case channel selectivity was not assessed.

The role of SARS E protein in calcium transport is another source of confusion in the field. Ca^2+^ fluxes through the SARS-1 E channel have been related to pathogenicity via activation of the NOD-, LRR- and pyrin domain-containing protein 3 (NLRP3) inflammasome [10], similar to observations in encephalomyocarditis virus viroporin 2B [29]. The three orders of magnitude difference in Ca^2+^ concentration between cytosol (~100 nM) and ERGIC lumen (~0.4 mM) [30,31] has been used to hypothesize that the E channel could compensate this difference based on its ERGIC localization [6,32]. However, conductance and selectivity of the E channel towards Ca^2+^ ions depend on the concentration of other monovalent ions. Indeed, the ionic transport properties of the SARS-1 E channel in solutions containing KCl and CaCl_2_ are strongly dependent on membrane lipid composition (especially on net charge), the ratio between these two salts and the total salt concentration [22]. This is known in the literature as anomalous mole fraction (AMFE) and is also observed in *bona fide* calcium selective and other nonspecific channels [33,34]. Therefore, rather than E protein being a calcium-specific channel, it probably affects calcium fluxes nonspecifically in the context of large alterations of ion homeostasis.

Herein, we performed planar membrane electrophysiology experiments and evaluated SARS-2 E channel selectivity under different conditions. We present evidence that E protein forms voltage-dependent ohmic channels, where low selectivity for anions or cations depends on the salt and lipid composition used. We use these data to discuss previous experiments, trying to reconcile the apparently contradictory notion of an unspecific proteolipidic channel—obtained from electrophysiology—with the structural view of a channel formed exclusively by pentamers. Regarding the latter, we performed new sedimentation velocity experiments involving E-TM, E-TR and E-FL in detergent. These experiments are consistent with pentamer formation, but the behavior of E protein suggests other forms of aggregation.

## 2. Results

### 2.1. SARS-2 E Is a Voltage-Dependent Ohmic Channel That Is Not Activated by Voltage

**Figure 2:**
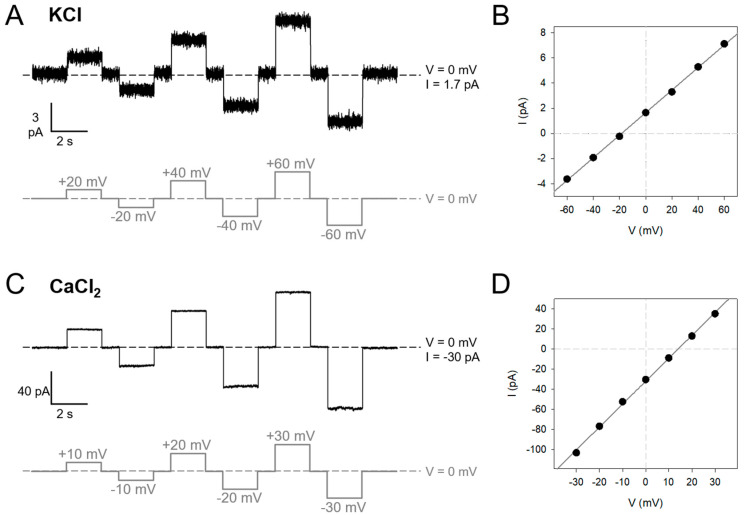
**Ohmic character of SARS-2 E-FL in KCl and CaCl_2_.** Example of stable current traces (**A**,**C**) and corresponding I–V curves (**B**,**D**) for SARS-2 E-FL channels in 500/50 mM KCl at the applied voltages shown in gray below each current trace; (**C**,**D**) same as (**A**,**B**) but using 500/50 mM CaCl_2_. The solid line in (**B**,**D**) is a linear fit to the experimental data.

Experiments with E-TM [11] and SARS-2 E-FL (Figure 2) show that channel activity is not voltage-activated, despite claims of the existence of a threshold voltage for ion conduction [28]. The current traces in Figure 2 were recorded under asymmetric salt conditions (500/50 mM) because a salt concentration gradient favored insertion of stable channels. However, similar events have also been obtained in symmetric conditions [11,35]. In KCl (Figure 2A,B), the current–voltage (I–V) curves generated (Figure 2B) were consistent with a purely ohmic behavior, and the same result was obtained using CaCl_2_ (Figure 2C,D). Note that the I–V curves display a nonzero current at zero applied voltage because of the use of asymmetric salt concentrations. These experiments demonstrate that SARS-2 E channel is not a voltage-activated channel, but currents show an ohmic behavior. These experiments, and previous assays that used SARS-1 E-FL protein [6,10,11,12], were performed at neutral pH. Therefore, in contrast to previous suggestions [3,18], the E channel is fully functional at neutral pH and neither acidification nor Ca^2+^ ions are necessary for activation. Low pH may modulate SARS-2 E-FL channel properties, but this investigation is out of the scope of the present study.

### 2.2. SARS-2 E Channels Are Mildly Selective but Strongly Modulated by Lipid Charge

**Figure 3:**
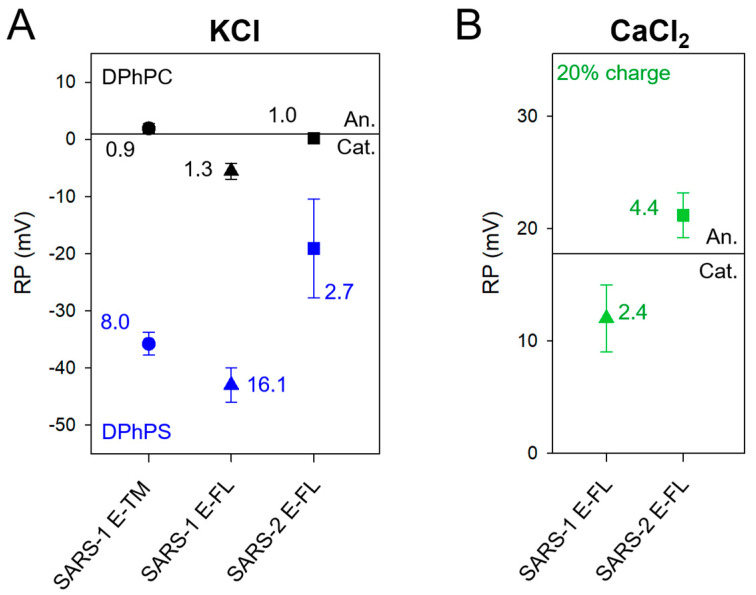
**Selectivity (RP) of E protein in different lipid compositions.** (**A**) RP measured in KCl with E-TM 7–38, SARS-1 E-FL or SARS-2 E-FL in neutral DPhPC (black) or negatively charged DPhPS (blue) membranes; (**B**) RP measured in CaCl_2_ solutions with SARS-1 E-FL or SARS-2 E-FL in membranes with 20% negatively charged lipids (ERGIC lipid composition). Numbers represent permeability ratios, P_+_/P_−_ (**A**) or P_−_/P_+_ (**B**). The horizontal lines indicate the RP of a neutral channel (V_diff_ = 0.93 mV, which corresponds to P_+_/P_−_ = 0.95 (**A**) or V_diff_ = 17.8 mV corresponding to P_−_/P_+_ = 3.49 (**B**)). The concentration gradient was 500/50 mM in all selectivity measurements and all RP values were corrected using the corresponding liquid junction potential from Henderson’s equation to eliminate the contribution of the electrode’s agarose bridges [27]. Number of experiments was *n* = 20 (black circle), 15 (black triangle), 16 (black square), 10 (blue circle), 10 (blue triangle), 26 (blue square), 10 (green triangle) and 19 (green square). Error bars indicate ± standard deviation. Data for SARS-1 were reported in [10,11].

To assess if the SARS-2 E channel is anion- or cation-selective, we performed experiments with a KCl or CaCl_2_ concentration gradient (500/50 mM) across the membrane and measured the reversal potential (RP) [36], i.e., the voltage required to yield zero current under a salt transmembrane gradient. For 500/50 mM CaCl_2_ and a neutral channel, RP is nonzero. It is ~17.8 mV due to the different bulk diffusion coefficients of Ca^2+^ and Cl^−^ ions. If the channel is selective, it departs from this value. In contrast, K^+^ and Cl^−^ diffusion coefficients are almost equal; therefore, for 500/50 mM KCl, the RP for a neutral channel is almost zero (V_diff_ = 0.93 mV using bulk ion activities). Thus, under the salt concentration gradient direction used in our experiments and the configuration of our setup (500 mM *cis*/50 mM *trans*, ground at *trans*), RP > V_diff_ corresponds to anion-selective channels. RP values were extracted from the x-intercept of the best linear fit of I–V plots like those shown in Figure 2B,D. When an I–V curve could not be obtained, the RP was determined by manually zeroing the current in the system. First, experiments were performed with KCl in neutral or negatively charged lipid membranes (Figure 3A).

Results with SARS-2 E-FL were compared with previous data obtained with E-TM and SARS-1 E-FL [11]. In neutral membranes (black symbols in Figure 3A) all samples were nonselective, with RPs values close to V_diff_ (solid line). These RPs are equivalent to permeability ratios P_+_/P_−_ [36] close to 1. However, in negatively charged membranes (DPhPS) the channels were clearly cation-selective, with permeability ratios P_+_/P_−_ ~ 3–16. That is, in negatively charged membranes, E channels allow for the passage of 3–16 K^+^ ions for each Cl^−^ ion.

SARS-2 E-FL selectivity was also assessed in CaCl_2_ (Figure 3B) and compared with that of SARS-1 E-FL [10]. SARS-2 E-FL in CaCl_2_ solutions showed slight anionic selectivity, with RPs equivalent to a permeability ratio P_−_/P_+_ of 4.4. That is, SARS-2 E-FL in CaCl_2_ allows for the passage of 4.4 Cl^−^ ions for each Ca^2+^ ion. Note, however, that due to the different diffusion coefficients of Ca^2+^ and Cl^−^ ions, a nonselective channel would yield RP = 17.8 mV, corresponding to P_−_/P_+_ = 3.5. Thus, the anionic preference of the protein channel itself is very small. This small anionic selectivity in SARS-2 E-FL (Figure 3B) contrasts with the mild preference for cations in SARS-1 E-FL (P_−_/P_+_ = 2.4) in the same salt conditions [10].

### 2.3. SARS-2 E Oligomerization in Detergent

**Figure 4:**
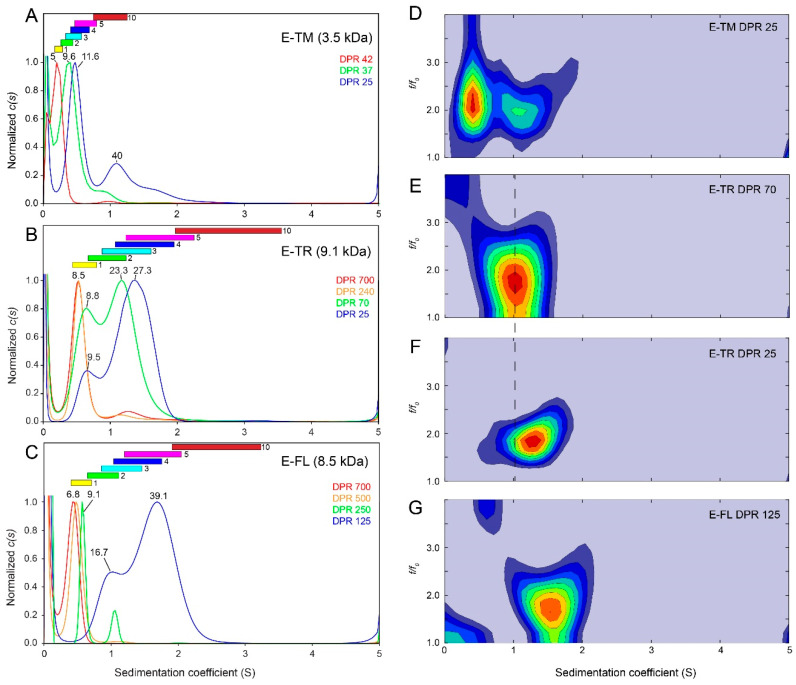
**AUC-SV profile of E protein in C14SB detergent micelles.** Comparison of *c*(*s*) size distribution of (**A**) E-TM, (**B**) His-E-TR and (**C**) E-FL at the indicated detergent-to-protein ratios (DPR, right). The predicted protein molecular weight according to SEDFIT using a common *f*/*f*_0_ for all species is shown with a number (kDa) above each band. Alternatively, the predicted range of S values for various n-oligomeric states (n is shown on the right of the bar) are shown as colored bars. These ranges were calculated as described in the Section 4; (**D**–**G**) two-dimensional plots *c*(*s*, *f*/*f*_0_) corresponding to E-TM (**D**), E-TR (**E**,**F**) and E-FL (**G**) at the DPRs indicated.

To test if the pentameric oligomerization is unique or involves other forms of assembly, we examined the three species shown in Figure 1 (E-TM, His-E-TR and E-FL) using sedimentation velocity (SV) experiments. The type of protein oligomer represented in the SV data was obtained in two ways: (i) using a common frictional ratio for all bands, the molecular weight was estimated from within SEDFIT, and (ii) predicting a range of possible S values for each type of oligomer in each of the species tested (Table 1 and Section 4). We found that the two methods were in general agreement. For the E-TM sample (monomer 3.5 kDa), the *c*(*s*) plot produced a sharp predominant band consistent with a rather homogeneous sample. When the peptide was more diluted (DPR 42), the S value was clearly consistent with a monomeric or dimeric form (Figure 4A) with an expected molecular weight of 5 kDa.

Increasing the peptide concentration (to lower DPR), the main single band shifted to higher S values, and at the lowest DPR ratio tested (DPR 25) the predicted molecular weight was 11.6 kDa (*n* ~ 3.3). This is consistent with the ranges of possible S values calculated for trimers and tetramers. We note that a single band with a concentration-dependent S value represents a rapid equilibrium between two unobservable species: possibly between monomers and a pentamer. The band would eventually shift towards the pentamer, but DPR could not be lowered further because of solubility issues. Interestingly, at DPR 25, small bands corresponding to larger oligomers (>1 S, MW ~ 40 kDa, equivalent to ~2 pentamers) can be observed. The 2D *c*(*s*, *f*/*f*_0_) plot (Figure 4D) also shows two densities, one for the intermediate form and the other to the larger aggregates. Although the observation of these larger aggregates could be due to detergent-dependent artifacts, this experimental condition (DPR 25) is comparable to the lipid-to-protein ratio (LPR) used in solid-state NMR (LPR 25), where data were consistent with interaction between E-TM pentamers [13]. Overall, the data in detergent and in lipid membranes suggest a dynamic exchange between monomers and a larger oligomer, possibly a pentamer. Once pentamers form, they tend to form larger assemblies.

The behavior of E protein was also tested using an extended peptide, 8–65 (His-E-TR in Figure 1, of 9.1 kDa). When diluted (DPR 700 and 240), it also showed a single sharp band consistent with a monomer (8.5 kDa, *n* = 0.93, and see yellow bar) (Figure 4B). Bands became much broader at higher concentrations (at DPR 70, bands at ~0.65 S and ~1.2 S) consistent with monomer (8.8 kDa, *n* = 0.96, and yellow bar) and dimer/trimer (23.3 kDa, *n* = 2.56, green/cyan bars) (Figure 4B). The larger band shifted with higher concentration, reaching 27.3 kDa (*n* = 3, cyan bar) at DPR 25. As in the case of E-TM, the shift in this band suggests an intermediate species between small (monomer?) and larger oligomers (pentamers?). The smaller band (at 8.8 kDa or 9.5 kDa) was reduced in intensity with decreasing DPR, but it did not shift. Therefore, it is likely to represent a real species, in this case a monomer. In contrast to E-TM, His-E-TR did not show larger aggregates (*n* > 5) at high concentration, possibly because of the presence of a significant part of the C-terminal domain (residues ~38–65), which may prevent interaction between pentamers. Since the bands are broader here, they could not be well resolved in the 2D plots (Figure 4E,F).

Finally, sample E-FL was tested at lower concentrations than in the other two samples because of its tendency to aggregate and precipitate. At DPR 700, 500 or 250, the band in the *c*(*s*) plot was sharp and consistent with monomers (Figure 4C). However, even at DPR 125, it formed oligomers (bands at ~1 S and ~1.75 S). The first band is consistent with dimers, whereas the larger band is consistent with pentamers (*n* = 4.6). The stronger tendency of E-FL to form oligomers compared to E-TR or E-TM may be caused by the presence of interlocked β-strands at the C-terminal domain, which may be especially effective in E-FL [14,21]. At this concentration, no larger aggregates similar to those observed for E-TM were observed. As in E-TR, the large width of the bands at higher concentration suggests a heterogeneous population when oligomers are formed. This results in a loss of resolution in the 2D plots (Figure 4G), although the mild split in density indicates the presence of two populations.

## 3. Discussion

Our electrophysiology experiments are consistent with SARS-2 E and SARS-1 E behaving as multi-ionic channels that allow for a rather indiscriminate passage of cations and anions without noticeable current rectification and full functionality at neutral pH.

SARS-2 E channel selectivity is strongly dependent on the lipid composition of the membrane. Taking the channel selectivity in a neutral membrane (pure DPhPC) as reference, RP measurements (Figure 3A, black symbols) show that both SARS-2 E and SARS-1 E channels are almost nonselective, but in negatively charged membranes (DPhPS) they show a clear preference for cations. This proves that lipid charge strongly modulates the channel preference for ions. Additionally, it supports the hypothesis that lipid molecules are part of the channel architecture, with some polar lipid headgroups facing the pore lumen. This proteolipidic character is also found in other viroporins [37,38] and was already suggested for SARS-1 E-FL and E-TM [11,12,23]. In contrast to the SARS-1 E channel, the selectivity of the SARS-2 E channel in CaCl_2_ salt and membranes with 20% of negatively charged lipids (like ERGIC membranes) is slightly anionic, which challenges the notion that the E protein could always act as a cationic channel that totally excludes anions, even less like a calcium channel that specifically selects Ca^2+^ over other cations. In this sense, the small (a few mV) differences between RP measured in 500/50 mM CaCl_2_ solutions and V_diff_ must be interpreted with caution given the uncertainty in the calculation of V_diff_. The latter uses bulk diffusion coefficients instead of the—unknown—real ones. Further, it does not take into account hydrodynamic constraints of nanoscale confinement and the tight interaction between calcium and lipids or protein residues [22,27]. In any case, the picture that emerges is that of an almost nonselective channel that exerts very minor, if any, discrimination between cations and anions.

Interestingly, selectivity of SARS-2 E-FL is slightly more anionic than SARS-1 E-FL for both charged lipid compositions and also for monovalent and divalent salts. This could be related to small differences in the C-terminal domain, where a negative glutamic acid of SARS-1 (E69) is replaced with a positive lysine (K70) in SARS-2. If that was the case, it would be expected that the ion selectivity of E-TM (identical for SARS-1 and SARS-2) in charged membranes would fall between that of SARS-1 E-FL and SARS-2 E-FL. This is indeed the case, and points to a subtle involvement of the C-terminal domain in the modulation of channel function.

Overall, the SARS-2 E channel cannot be considered a *bona fide* calcium channel in the same sense as other channels that strongly select calcium over other cations (e.g., calcium channels select for Ca^2+^ over Na^+^ with a ratio > 1000:1) [36]. On the contrary, electrophysiology yields a picture of a proteolipidic nonspecific channel. The question that arises is how these data can be reconciled with the view of a pentamer with possibly a highly specific narrow path for ions [3,13,18]. Although AUC experiments with E-FL show that a pentamer is the most likely oligomeric size, the rapid equilibrium observed between monomers and larger oligomers, and the evident aggregation behavior observed for E-TM, could explain electrophysiology results. In the latter technique, the lipid-to-protein ratio cannot be tightly controlled; assuming complete incorporation of E protein in the BLMs, and no lipid losses, experiments reported here would correspond to an LPR of ~500, which would result in monomers without channel activity according to the SV results we obtained in detergent. Thus, the affinity between monomers, including that of E-TM, must be considerably higher in the lipid membrane, although still subject to a dynamic exchange between monomers and oligomers.

The variability of conductances reported in earlier studies [10,11,12,22] (where a histogram of currents was the only way to obtain an average conductance value), and the large error bars obtained in some of the experiments reported here, point to a multiplicity of structures rather than a fixed one. This multiplicity may be represented by pentameric forms that have different lipid complement, a rapid exchange between monomers and pentamers or other species, or a formation of larger complexes, as shown by our SV results. Different oligomeric configurations would yield slightly different channel selectivities, although what is captured in our experiments can be considered an average of this hypothetic heterogeneous population. This view would be consistent with the observed lipid-dependent selectivity [12], which can render the channel cationic or anionic, as this is only possible if selectivity is very low in the first place. Although the presence of charged lipids shifts the selectivity towards a cationic one, these values are still far from those observed in highly specific channels [36].

Despite the dynamic behavior of the E protein constructs tested, we stress that our SV experiments do not contradict the view that E protein forms pentamers in detergent and in membranes. This is especially evident in the case of E-FL, which readily formed pentamers even in relatively diluted conditions. In the case of E-TM and E-TR, however, affinity was lower, and formation of 100% pentameric population in detergent requires a high peptide concentration. However, the affinity constant may be higher in lipids.

In contrast to ssNMR, AUC allows testing of a range of detergent-to-protein ratios, including diluted conditions where E protein is monomeric. When concentration increases, the peptide tends to form larger oligomers. In the past, we performed experiments using E-FL, E-TR or E-TM in equilibrium sedimentation, and the best fit to the data was a model that involved monomers and pentamers. In sedimentation velocity (shown in the present paper) it is not possible to fit the data to a certain equilibrium model. What we observe instead is different species present in the sample if equilibrium is slow (e.g., monomers and pentamers). If there is a rapid equilibrium, as is the case here, we only see an intermediate species that shifts to higher molecular weight with increasing concentration. We believe that at higher concentration than the one we tested for E-TM and E-TR, we would directly observe pentameric oligomers in detergent, but solubility issues prevented this. However, consistent with previous ssNMR results, we observe that E-TM at high peptide concentration (low DPR) has a tendency to form larger oligomers than pentamers, perhaps by aggregation of two or more pentamers. This may be an artifact caused by using just the TM domain. Alternatively, it may be present in E-FL in cellular membranes, where high protein densities can be reached if the protein is attracted to regions of certain curvature (convex or concave) or to certain lipid compositions (e.g., rafts). These larger aggregates may be linked to functions unrelated to ion channel activity, such as membrane fusion or interaction with other viral or host proteins.

In conclusion, our results do not support that the SARS E protein is cation-selective, in the sense of fully excluding anions, and even less that it is calcium-specific. The modulation observed after modification of the membrane charges suggests a population of channels with overall near nonselective ion activity, which is easily tipped to one side (cationic) or the other (anionic) depending on the environment. It is also possible that the population is heterogeneous and dynamic, as suggested by the large width of conductance histograms and the multiple oligomeric forms observed in SV experiments. Accordingly, the E channel should affect the general ion homeostasis of the infected cell, rather than being calcium-specific, although release of calcium from ERGIC is one of the possible outcomes.

Our work presents evidence supporting the role of SARS-CoV E as a proteolipidic channel with weak selectivity and heterogeneous oligomerization and provides clues to reconciling the apparently contradictory notion of an unspecific proteolipidic channel obtained from electrophysiology with the structural view of a channel formed exclusively by pentamers. The two main findings of this work, the weak channel selectivity and the heterogeneous oligomerization, suggest further work in two directions: First, clarifying the functional role of the E channel at the ERGIC membrane and whether altering ion homeostasis is essential for virulence or otherwise its permeability to other small molecules that trigger the inflammation process. Second, exploring how protein–lipid ratios determine channel conformation and function, aiming to reconcile the (narrow pore) high-resolution pentameric 3D structures available with the electrophysiology measurements. Only detailed and robust knowledge of E channel function can be the starting point for future research focusing on antiviral drugs targeting the SARS-CoV-2 E protein.

## 4. Materials and Methods

### 4.1. E Protein Synthesis, Expression, and Purification

The transmembrane domain of SARS-2 E corresponding to residues 7–38 (E-TM) was synthesized using solid-phase peptide synthesis and purified as described previously [11]. The truncated SARS-2 E protein corresponding to residues 8–65 with N-terminal 6-His tag (6H-E-TR) was expressed and purified as described previously [21]. The full-length SARS-2 E protein (E-FL) was cloned into pET28b plasmid, with a 6-His tag followed by a YbeL expression tag [39] and a TEV protease cleavage site added to the N-terminus of E-FL sequence, resulting in 6-His_YbeL_TEV_E-FL construct. *E. coli* BL21-CodonPlus(DE3)-RIPL (Agilent, Santa Clara, CA, USA) competent cells were transformed with the plasmid, grown in ZYM-505 media [40] at 37 °C, induced overnight with 1.0 mM IPTG at 30 °C, and harvested using centrifugation. The fusion protein was isolated as inclusion bodies and refolded from urea into 50 mM Tris-HCl pH 8, 150 mM NaCl, 1 mM EDTA and 0.1% (*v*/*v*) N,N-Dimethyldodecylamine N-oxide (LDAO, Sigma, St. Louis, MO, USA) using dialysis. The YbeL tag was cleaved by incubation with TEV protease (1:50 molar ratio) and the mixture was TCA-precipitated and lyophilized. The lyophilized mixture was redissolved in 1% (*v*/*v*) TFA in ACN, injected into a Jupiter 5 μm C4 300 Å 250 × 10 mm reversed-phase column (Phenomenex, Torrance, CA, USA), and eluted using linear IPA/ACN (4/1 with 0.1% TFA *v*/*v*) gradient. Fractions containing E-FL were identified using SDS-PAGE and MALDI-TOF/MS, pooled and lyophilized in 50% (*v*/*v*) ACN, 1 mM HCl to remove residual TFA.

### 4.2. Analytical Ultracentrifugation Sedimentation Velocity (AUC-SV)

AUC-SV experiments were performed using a ProteomeLab XL-I Analytical Ultracentrifuge (Beckman Coulter). Samples for AUC-SV were prepared by dissolving lyophilized peptides in 50 mM Tris-HCl pH 7.3, 100 mM NaCl, 5 mM 3-(N,N-Dimethylmyristoylammonio) propanesulfonate (C14SB, Sigma) and 29.4% (*v*/*v*) D_2_O (99.9%, Cambridge Isotope Laboratories, Tewksbury, MA, USA). Samples were loaded into 2-channel AUC cells with 12 mm Epon centerpiece and quartz windows and centrifuged at 50,000 rpm at 20 °C. Absorbance scans at 230 or 280 nm were collected every 5 min for up to 15 h. The data were analyzed using the *c*(*s*) and *c*(*s*, *f*/*f*_0_) size distribution models in SEDFIT [41] and visualized in GUSSI [42]. The range of S values for different E protein oligomers in C14SB micelles was calculated as described previously [43]. The MW and partial specific volume of E-TM, E-TR and E-FL were calculated using SEDNTERP [44] (Table 1). The range of frictional ratios used for the calculation were based on the values obtained from *c*(*s*, *f*/*f*_0_) model fitting in SEDFIT for the more diluted and the most concentrated sample. Buffer density and viscosity was 1.0353 g/mL and 1.0997 cP, respectively, calculated using SEDNTERP [44], with ±3% variation in D_2_O concentration included in the S value prediction range and an interval ῡ for the detergent of 0.965–0.978 mL/g, based on values from the literature [45] and our own density-matching data.

### 4.3. Planar Membrane Formation and Ion Channel Reconstitution

1,2-diphytanoyl-sn-glycero-3-phosphocholine (DPhPC), 1,2-dipalmitoyl-sn-glycero-3-phospho-L-serine (DPhPS), 1,2-dioleoyl-sn-glycero-3-phosphocholine (DOPC), 1,2-dioleoyl-sn-glycero-3-phosphoethanolamine (DOPE), 1,2-dioleoyl-sn-glycero-3-phospho-L-serine (DOPS), 1,2-dioleoyl-sn-glycero-3-phospho-(1′-myo-inositol) (DOPI) and plant-derived cholesterol (Chol) were purchased from Avanti Polar Lipids (Alabaster, AL, USA). Planar bilayers were formed by apposition of two monolayers prepared from a solution at 5 mg/mL in pentane of pure lipids (DPhPC or DPhPS for E-TM, SARS-1 E-FL and SARS-2 E-FL in KCl) or lipid mixtures with 20% charge (DOPC:DOPE:DOPS 59:24:17 (mol/mol) for SARS-1 E-FL in CaCl_2_ or DOPC:DOPE:DOPS:DOPI:Chol 45:20:13:7:15 (mol/mol) for SARS-2 E-FL in CaCl_2_). Lipids were added on ~150 μm diameter orifices in a 15 μm thick Teflon partition that separated two identical chambers [46,47]. The orifices were pretreated with a 1% solution of hexadecane in pentane. Aqueous solutions were buffered with 5 mM HEPES, and the pH was fixed at pH = 6 by adding HCl, KOH or Ca(OH)_2_ and controlled during the experiments with a GLP22 pH meter (Crison, Singapore). All measurements were performed at room temperature (23 ± 1 °C). Ion channel insertion was achieved by adding up to 5 μL of a 300 μg/mL solution of SARS-2 E-FL protein in ethanol to one side of the chamber (*cis* side).

### 4.4. Ionic Current Recording and Selectivity Measurements

An electric potential was applied using Ag/AgCl electrodes in 2 M KCl, 1.5% agarose bridges assembled within standard 250 μL pipette tips. The potential was defined as positive when it was higher on the side of protein addition (*cis* side), whereas the *trans* side was set to ground. An Axopatch 200B amplifier (Molecular Devices, Sunnyvale, CA, USA) in voltage-clamp mode was used to measure the current and the applied potential. Data were filtered by an integrated lowpass 8-pole Bessel filter at 10 kHz, saved with a sampling frequency of 50 kHz with a Digidata 1440A (Molecular Devices, Sunnyvale, CA, USA) and analyzed using pClamp 10.7 software (Molecular Devices, Sunnyvale, CA, USA). For visualization, current traces were digitally filtered at 100 Hz using a lowpass Bessel (8-pole) filter. The chamber and the head stage were isolated from external noise sources with a double metal screen (Amuneal Manufacturing Corp., Philadelphia, PA, USA). The reversal potential (the voltage corresponding to zero current) was measured on one or several channels under a salt concentration gradient of 500/50 mM KCl or CaCl_2_. The measured value was corrected using the liquid junction potential from Henderson’s equation [27] to obtain the final reversal potential. Permeability ratios between Ca^2+^ and Cl^−^ or K^+^ and Cl^−^, were calculated according to the Goldman–Hodgkin–Katz equation [36] using the corresponding ion activities.

## Figures and Tables

**Table 1:** **Predicted range of sedimentation coefficients (S) for each oligomer.** Monomeric molecular weight, partial specific volumes (ῡ) and range of frictional ratios used to determine a safe range of expected S values for each oligomer type corresponding to E-TM, E-TR and E-FL in C14SB detergent micelles. As in our previous paper [14], the S-value range calculation also assumed a ±3% error in the D_2_O concentration (i.e., 26–32% *v*/*v*). The possible range of frictional values was obtained from the analysis *c*(*s*, *f*/*f*_0_) of different concentrations of a sample and used conservative estimates of minimum and maximum *f*/*f*_0_ values observed for the main densities in *c*(*s*, *f*/*f*_0_) plots at the lowest and highest DPR tested. For example, for E-FL, the minimum ratio was 1.5 and the maximum was 2.5 (see Figure 4D–G). The lower part of the Table shows the calculated range of S values as minimum–maximum and median value (within parentheses).

Protein-Specific Parameters			
	E-TM	6H-E-TR	E-FL
Monomeric MW (Da)	3490	9081	8542
ῡ (20 °C) (mL/g)	0.7929	0.7549	0.7656
Frictional ratio, *f*/*f*_0_	1.8–3.0	1.5–2.7	1.5–2.5
**Predicted S-Value Range**			
	**E-TM**	**6H-E-TR**	**E-FL**
Monomer (S)	0.16–0.27 (0.21)	0.42–0.76 (0.56)	0.41–0.69 (0.55)
Dimer (S)	0.25–0.43 (0.34)	0.66–1.22 (0.93)	0.65–1.11 (0.87)
Trimer (S)	0.33–0.57 (0.45)	0.87–1.60 (1.22)	0.85–1.45 (1.14)
Tetramer (S)	0.40–0.69 (0.54)	1.05–1.93 (1.48)	1.03–1.76 (1.38)
Pentamer (S)	0.47–0.80 (0.63)	1.22–2.24 (1.72)	1.19–2.04 (1.60)
Decamer (S)	0.74–1.27 (1.00)	1.94–3.56 (2.73)	1.90–3.23 (2.55)

## Data Availability

Not applicable.
